# Network Pharmacology and Molecular Docking Reveal Anti-Asthmatic Potential of *Zephyranthes rosea* Lindl. in an Ovalbumin-Induced Asthma Model

**DOI:** 10.3390/ph17111558

**Published:** 2024-11-20

**Authors:** Amir Ali, Hafiz Majid Rasheed, Siddique Akber Ansari, Shoeb Anwar Ansari, Hamad M. Alkahtani

**Affiliations:** 1 Faculty of Pharmacy, The University of Lahore, Lahore 54590, Pakistan; 2 Department of Pharmaceutical Chemistry, College of Pharmacy, King Saud University, P.O. Box 2457, Riyadh 11451, Saudi Arabia; sansari@ksu.edu.sa (S.A.A.); ahamad@ksu.edu.sa (H.M.A.); 3 Department of Drug Science and Technology, University of Turin, 10124 Turin, Italy; shoeb.ansari@edu.unito.it

**Keywords:** asthma, ovalbumin, bronchoalveolar lavage fluid, *interleukin 6*, AKT serine/threonine kinase 1, proto-oncogene tyrosine-protein kinase Src, network pharmacology, tumor necrosis factor alpha, *Zephyranthes rosea* bulb

## Abstract

**Background:** This study aimed to evaluate the anti-inflammatory effects of a *Zephyranthes rosea* in an ovalbumin-induced asthma model. **Methods:** Allergic asthma was induced in mice via intraperitoneal injection, followed by intranasal ovalbumin challenge. Methanolic extract of *Z. rosea* bulb was orally administered to asthmatic mice for 14 days. Hematological parameters for bronchoalveolar lavage fluid (BALF) and blood were analyzed. The mRNA expression levels of interleukins and transforming growth factor beta (*TGF-β1*) in lung tissues were determined using reverse transcriptase–polymerase chain reaction (RT–PCR). Network pharmacology analysis was used to find possible *Z. rosea* targets. After building a protein–protein interaction network to find hub genes, GO and KEGG enrichment analyses were carried out to determine the potential mechanism. In silico analysis was performed by Molecular Operating Environment. **Results:** GC-MS analysis of *Z. rosea* extract detected major classes of phytochemicals. Hematological parameters in blood and BALF from *Z. rosea* extract-treated animals were significantly reduced in a dose-dependent fashion. Histopathology revealed that *Z. rosea* bulb had an ameliorative effect on lung tissues. Moreover, treatment with *Z. rosea* bulb extract significantly restored the normal levels of *IL-4*, *IL-6*, *IL-1β*, *IL-10*, *IL-13*, and *TGF-β1* in allergic asthmatic mice compared to the diseased group. In silico analysis, particularly of the binding affinities of *Z. rosea* bulb phytoconstituents for *IL6*, *AKT1*, and *Src*, supported in vivo results. **Conclusions:** These findings indicated that *Z. rosea* bulb extract significantly ameliorates cellular and molecular biomarkers of bronchial inflammation and could be a potential candidate for treating allergic asthma.

## 1. Introduction

The word “asthma” is Greek in origin and means “breathlessness”. Over the past few years, there has been an increase in asthma-related mortality and morbidity [1]. Airway inflammation, edema, and obstruction are the three main classifications of asthma [2]. Numerous studies have demonstrated the critical role of T lymphocytes, eosinophils, neutrophils, macrophages, and epithelial cells in the pathophysiology of allergen-induced asthma [3,4,5,6]. Various cytokines are known to affect airway inflammation [7,8], which may arise as a result of an excess of Th2-mediated cytokines, produced due to an imbalance between Th1- and Th2-mediated pathways [9]. The Th2-type cytokine *IL5* can regulate eosinophil development and differentiation and control eosinophils in allergic asthma [10]. Increased *IL5* levels have been observed in asthmatic patients with eosinophilic inflammation in earlier research [11,12]. By promoting Th0 differentiation in favor of Th2 cells, *IL4* affects the inflammatory process associated with allergic asthma [9]. Vascular cell adhesion can be induced on endothelial cells by *IL4* [13,14,15].

The pathogenesis of asthma involves a cascade of events triggered by various factors, including allergens, irritants, and infections. These factors initiate immune system activation and the subsequent release of inflammatory mediators such as histamine, leukotrienes, and cytokines. These cytokines, such as *IL-6*, *IL-1β*, and other proinflammatory cytokines, signal via receptors that activate tyrosine protein kinase (Janus kinase 2) upon ligand binding. This leads to the phosphorylation and activation of *STAT* proteins, which subsequently regulate the expression of inflammatory and remodeling genes.

Tumor necrosis factor alpha (*TNF-α*) induces the production of *IL-4* and *IL-13*, thereby amplifying the allergic response. *TNF-α* synergizes with *IL-6* to prolong eosinophil activation; hence, it induces the trafficking of adhesion molecules that support eosinophils into the lungs during asthma attacks [16]. *IL-6* contributes to airway inflammation by promoting the differentiation of Th2 cells and the production of Th2 cytokines such as *IL-4* and *IL-13*. These changes stiffen, narrow, and cause the airways to become hyperresponsive, worsening obstruction and difficulty breathing and resulting in asthma symptoms [17,18].

Historically, the first line of treatment for asthma has been inhaled β2 agonists. However, the use of these medicines has been restricted because of their numerous local and systemic side effects like muscle weakening and weight gain [19]. The effectiveness of treating allergic asthma with corticosteroids is limited due to adverse effects, including enlarged eyes, weight gain, a moon-like face, muscle weakness, decreased bone metabolism, stunted growth in children, and adrenal suppression. Due to these severe side effects, both patients and physicians seek alternative and/or traditional medicine [20,21].

The plants in the Amaryllidaceae family are monocotyledons with high ornamental, economic, and medical value [22]. The Amaryllidaceae family includes approximately 75 genera and 1100 species [23]. The genus *Zephyranthes* comprises largely perennial bulbous plants and includes approximately 90 species, but few of these species have been investigated for their chemical constituents. A wide variety of phytochemicals are found in this genus, among which alkaloids are particularly significant because of their pharmacological effects [24]. Several species have been assessed for their pharmacological activities.

*Zephyranthes rosea* Lindl. is a small perennial herbaceous bulbous plant of the Amaryllidaceae family [25]. It is synonymously known as *Amaryllis rosea*, *Amaryllis carnea*, *Zephyranthes carnea*, and *Atamasco rosea*. The plants are commonly called pink rain lily, rosy rain lily, Cuban zephyr lily, pink rain lily, rose zephyr lily, and rose fairy lily. *Z. rosea* is commonly found in Peru and Colombia. In Pakistan, it is available as an ornamental plant. It is extensively cultivated in temperate and tropical regions as an ornamental plant [26]. *Z. rosea* is a small perennial herbaceous monocot (5.9–7.9 in). These plants have 5–6 narrow, dark, green linear leaves (0.12–0.16 in width) with spherical tunicate bulbs (0.59–0.98 in width) [27,28]. The bulbs of *Z. rosea* are covered with a black tunica layer with contractile roots [29].

Many countries have utilized *Z. rosea* as a traditional medicine. In China, whole plants have been used to treat breast cancer. Extracts from the bulb of the plant have been utilized medicinally for a wide range of therapeutic applications, including diabetes treatment, viral infections, and ear and chest ailments [30]. The plant has two alkaloids that have been isolated: 4% galanthamine and 92% lycorine [31]. Four alkaloids have also been isolated from plant bulb extracts: crinamine, hemanthamine, maritidine, and (+)-epimaritidine [32]. *Z. rosea* has been reported to have antibacterial, antioxidant, anti-inflammatory, and antidiabetic activities.

These findings logically link the folkloric use of *Z. rosea* to chest ailments and inflammatory disorders. These results support the reliability of the traditional application of *Z. rosea* as a remedy for respiratory system disorders and warrant further scientific investigation of the anti-asthmatic activity of this plant. Therefore, this current study investigated the anti-asthmatic potential of *Z. rosea* in allergen-induced asthmatic mice.

## 2. Results

### 2.1. Extraction Yield

The extract of the *Z. rosea* bulb powder was obtained by cold maceration using commercial-grade methanol. The percentage yield of the *Z. rosea* extract was calculated, and the yield was 5.5%. The extract was dark brown in color, bitter in taste, semisolid in consistency, and slightly aromatic.

### 2.2. Phytocompounds from Z. rosea Bulb

**Figure 1:**
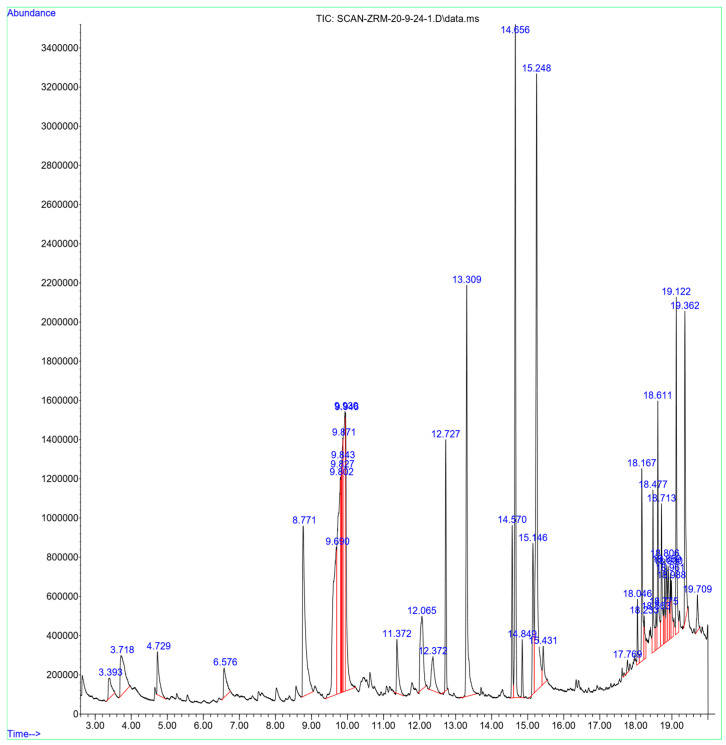
GC-MS chromatogram of the methanolic extract of *Z. rosea* bulb.

A Total of 38 Phytocompounds in the *Z. rosea* Bulb Were Identified by GC-MS Analysis (Figure 1). The major constituents were identified in the extract were 9,12-Octadecadienoic acid (Z,Z)- (11.19%), 10,13-Octadecadienoic acid, methyl ester (7.82%), 1-Isopropoxy-2,2,3-trimethyl aziridine (7.05%), and *n*-hexadecanoic acid (6.12%), and many other compounds were identified. The names of the compounds, retention time (mins), peak area, and peak area (%) are listed in Table 1. All 38 compounds were accepted by Lipinski’s rules (Molecular Weight ≤ 500 g/mol; Moriguchi octanol-water partition coefficient ≤ 4.15; Number of Nitrogen or Oxygen ≤ 10; Number of NH or OH ≤ 5), and all phytocompounds corresponded with the standard of “Abbott Bioavailability Score (>0.1)” through SwissADME. The TPSA (Topological Polar Surface Area) value of all phytocompounds was also accepted (Table 2).

### 2.3. Hematological Results

#### 2.3.1. Effect of *Z. rosea* Extract on Hematological Parameters in Blood

The results of hematological analysis revealed a decrease in Hb levels (g/dL) in disease control (13.21 ± 0.152) group animals compared to those in normal control (14.9 ± 0.058) and positive control (13.95 ± 0.08) group animals. However, treatment with *Z. rosea* 200 (12.85 ± 0.088), *Z. rosea* 400 (14.21 ± 0.12), or *Z. rosea* 600 (14.97 ± 0.088) increased the Hb level to a level comparable to that of the normal control. Similarly, there was a decrease in RBC density (×10^6^/µL) in disease control (7.21 ± 0.091) group animals compared to that in normal control (8.95 ± 0.117) and positive control (7.91 ± 0.216) group animals. However, treatment with *Z. rosea* 200 (7.29 ± 0.35), *Z. rosea* 400 (8.26 ± 0.05), or *Z. rosea* 600 (8.81 ± 0.03) increased the RBC count to a level comparable to that of the normal control. However, there were increases in platelet, lymphocyte, and monocyte counts in the disease control group animals compared to those in the normal control, positive control, and *Z. rosea* extract-treated animals. The results of these parameters are presented in Table S1.

#### 2.3.2. Effect of *Z. rosea* Extract on WBCs in BALF

The results of hematological analysis of BALF showed an increase in lymphocytes and monocytes (%) in disease control (72.67 ± 1.2; 4.72 ± 0.33) group animals compared to normal control (32.67 ± 0.88; 1.71 ± 0.33) and positive control (34.0 ± 0.58; 2.42 ± 0.33) group animals, respectively. Treatment with *Z. rosea* 200 (69.32 ± 0.88; 3.05 ± 0.33), *Z. rosea* 400 (59.31 ± 0.88; 2.92 ± 0.58), or *Z. rosea* 600 (57.62 ± 1.2; 2.79 ± 0.33) decreased the lymphocyte and monocyte counts to levels comparable to those of the normal and positive controls (Table 3).

#### 2.3.3. Effect of *Z. rosea* Extract on TLC in Blood and BALF

**Figure 2:**
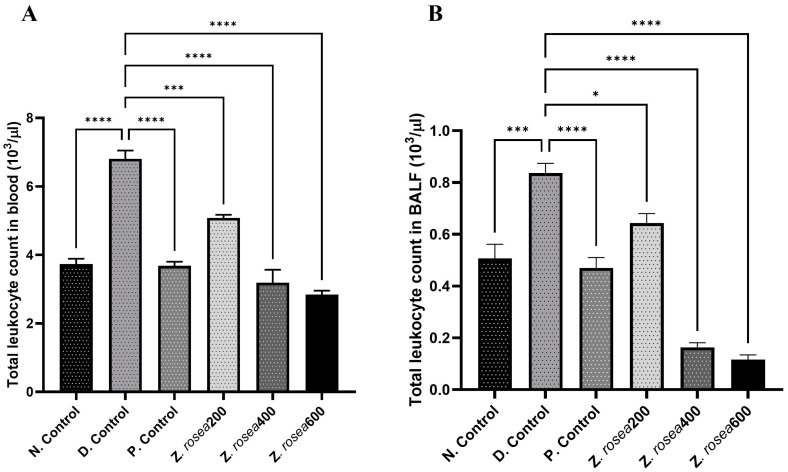
The effect of *Z. rosea* extract on blood (**A**) and BALF (**B**) TLC plates. Statistical analysis was made using one-way (ANOVA) followed by Dunnett’s post hoc analysis for multiple comparisons. Comparisons were considered significant at * *p* < 0.05, *** *p* < 0.001, and **** *p* < 0.0001, respectively, in contrast with the disease control group.

The data showed an increase in TLC (×10^3^/µL) in the blood of disease control (6.80 ± 0.420) group animals compared to that in the blood of normal control (3.73 ± 0.270) group animals. Treatment with *Z. rosea* 200 (5.08 ± 0.161), *Z. rosea* 400 (3.19 ± 0.656), *Z. rosea* 600 (2.84 ± 0.197), or methylprednisolone (3.68 ± 0.208) attenuated TLC in the blood (Figure 2A). Similarly, the results revealed an increase in TLC in the BALF of disease control (0.837 ± 0.064) group animals compared to that in the BALF of normal control (0.507 ± 0.095) group animals. Treatment with *Z. rosea* 200 (0.643 ± 0.064), *Z. rosea* 400 (0.163 ± 0.031), *Z. rosea* 600 (0.117 ± 0.03), or methylprednisolone (0.47 ± 0.07) attenuated TLC in the blood (Figure 2B).

#### 2.3.4. Effect of *Z. rosea* Extract on Neutrophils in Blood and BALF

**Figure 3:**
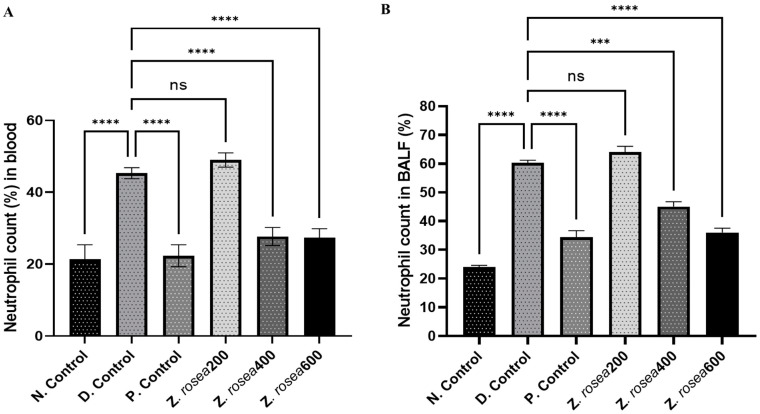
Effect of *Z. rosea* extract on neutrophils in blood (**A**) and BALF (**B**). Statistical analysis was made using one-way (ANOVA) followed by Dunnett’s post hoc analysis for multiple comparisons. Comparisons were considered significant at ns = non-significant, *** *p* < 0.001, and **** *p* < 0.0001, respectively, in contrast with the disease control group.

Neutrophils (%) were elevated in the blood of disease control (45.33 ± 1.528) group animals compared with those of normal control group (21.33 ± 4.04) animals. However, the neutrophil count was lower in *Z. rosea* 200 (49.0 ± 2.00), *Z. rosea* 400 (27.67 ± 2.52), *Z. rosea* 600 (27.33 ± 2.51), and methylprednisolone (22.33 ± 1.528)-treated animals than in disease control animals (Figure 3A). Similarly, the number of neutrophils was greater in the BALF of disease control (60.33 ± 1.528) group animals than in that of normal control group (24.0 ± 1.00) animals. However, the number of neutrophils was lower in *Z. rosea* 200 (64.00 ± 3.606), *Z. rosea* 400 (45.0 ± 3.00), *Z. rosea* 600 (39.00 ± 2.64), and methylprednisolone (34.33 ± 4.04)-treated animals than in disease control animals (Figure 3B).

#### 2.3.5. Effect of *Z. rosea* Extract on Eosinophils in Blood and BALF

**Figure 4:**
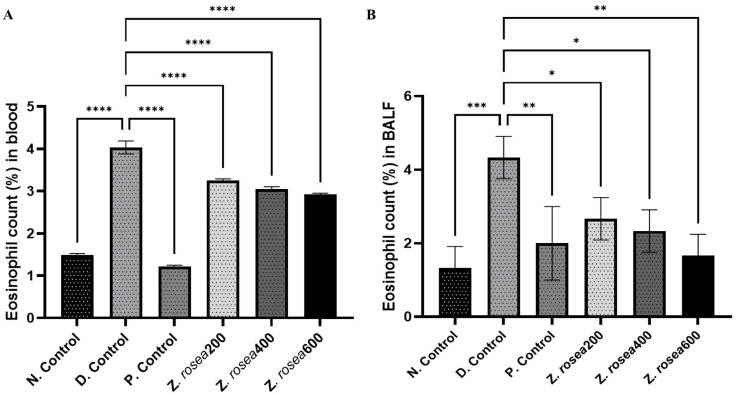
Effect of *Z. rosea* extract on eosinophils in blood (**A**) and BALF (**B**). Statistical analysis was made using one-way (ANOVA) followed by Dunnett’s post hoc analysis for multiple comparisons. Comparisons were considered significant at * *p* < 0.05, ** *p* < 0.01, *** *p* < 0.001, and **** *p* < 0.0001, respectively, in contrast with the disease control group.

Eosinophil counts (%) were elevated in the blood of disease control (4.0 ± 0.15) group animals compared with those of normal control (1.48 ± 0.375) animals. However, the number of neutrophils was lower in *Z. rosea* 200 (3.25 ± 0.40), *Z. rosea* 400 (3.04 ± 0.33), *Z. rosea* 600 (2.92 ± 0.03), and methylprednisolone (1.21 ± 0.03)-treated animals than in diseased control animals (Figure 4A). Similarly, the number of eosinophils was greater in the BALF of disease control (4.33 ± 0.58) group animals than in that of normal control group (1.33 ± 0.577) animals. However, compared with those in disease control animals, the number of neutrophils in *Z. rosea* 200 (2.67 ± 0.577), *Z. rosea* 400 (2.33 ± 0.58), *Z. rosea* 600 (1.67 ± 0.577), and methylprednisolone (2.00 ± 1.00) treated animals decreased (Figure 4B).

### 2.4. Histopathological Interpretation of Lung Tissues

**Figure 5:**
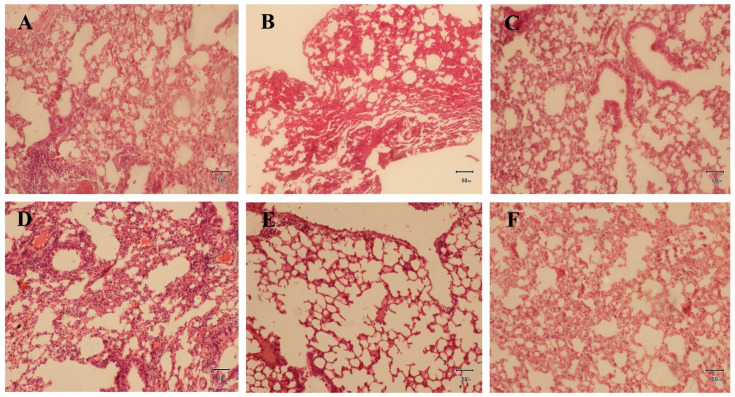
Representative histological microphotographs of lung tissues from the (**A**) normal control, (**B**) disease control, and control groups and from the (**C**) positive control, (**D**) *Z. rosea* extract 200, (**E**) *Z. rosea* extract 400, and (**F**) *Z. rosea* extract 600 groups.

Microscopic observation of the lung tissues revealed slightly collapsed alveoli with abundant infiltration of tissue (atelectasis) in the disease control group animals (Figure 5B). However, no tissue changes were observed in the normal control animals (Figure 5A). Similarly, treatment with *Z. rosea* bulb extract reduced the expression of the abovementioned histopathological features (alveolar collapse and tissue infiltration) in an apparent dose-dependent manner (Figure 5D–F). The beneficial effects on the lung tissues of *Z. rosea* 600-treated animals were comparable to those of standard drug (methylprednisolone)-treated animals (Figure 5C).

### 2.5. Effect of Z. rosea Extract on Proinflammatory Interleukins

**Figure 6:**
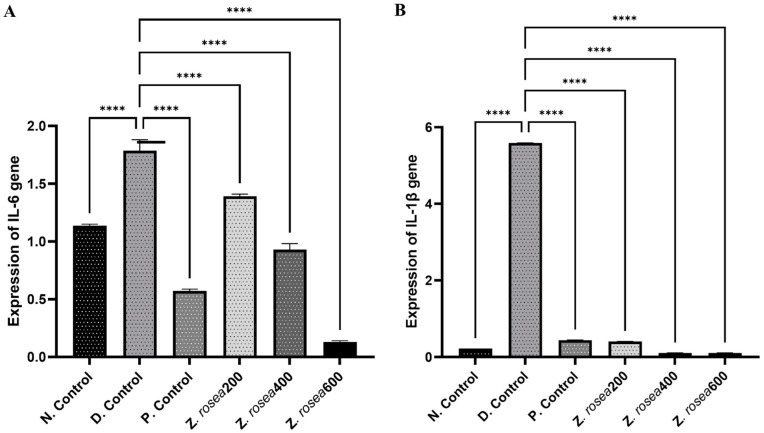
The effect of *Z. rosea* extract on the expression of (**A**) IL-6 and (**B**) IL-1β. Statistical analysis was made using one-way (ANOVA) followed by Dunnett’s post hoc analysis for multiple comparisons. Comparisons were considered significant at **** *p* < 0.0001, in contrast with the disease control group.

The levels of proinflammatory *interleukins* (*IL-6* and *IL-1β*) were measured using RT–PCR to evaluate the differences in the expression of inflammatory mediators among the groups. The levels of these interleukins were greater in disease control animals than in untreated animals and those treated with standard or *Z. rosea* extract. These results are given in Table 4 and Figure 6.

### 2.6. Effect of Z. rosea Extract on Anti-Inflammatory Interleukins

**Figure 7:**
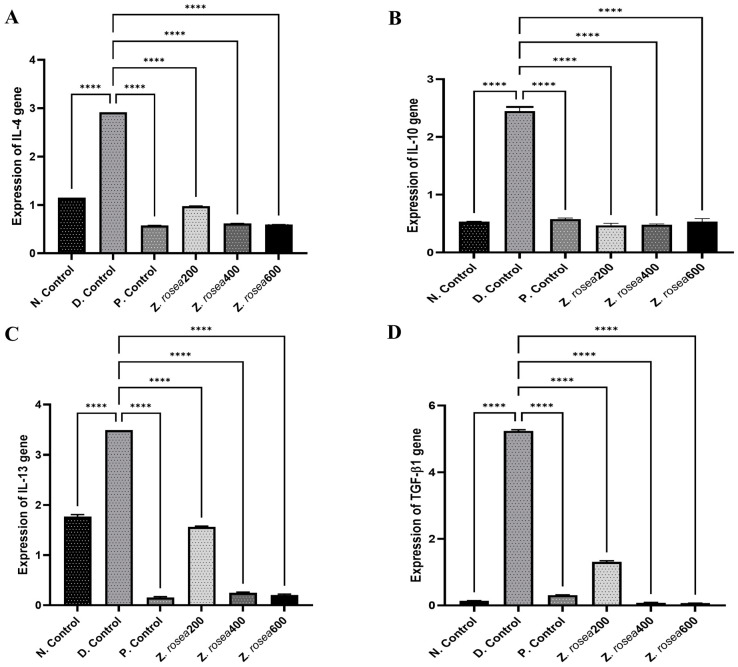
The effect of *Z. rosea* extract on the expression of (**A**) IL-4, (**B**) IL-10, (**C**) IL-13, and (**D**) TGF-β1. Statistical analysis was made using one-way (ANOVA) followed by Dunnett’s post hoc analysis for multiple comparisons. Comparisons were considered significant at **** *p* < 0.0001 in contrast with the disease control group.

The levels of anti-inflammatory interleukins (*IL-4*, *IL-10*, and *IL-13*) and *TGF-β1* were measured, and the levels of interleukins and *TGF-β1* were greater in disease control animals than in untreated animals and those treated with standard or *Z. rosea* extract. These results are presented in Table 5 and Figure 7.

### 2.7. Targets Associated with Phytocompounds

A total of 603 key targets were predicted from STP associated with 38 phytocompounds. Multiple targets were associated with each of these active molecules. This is a strong indication that many targets may induce a synergistic effect when *Z. rosea* bulbs serve as an anti-asthmatic agent.

### 2.8. Ovelapping Targets Between Allergic-Asthma-Associated Targets and Phytocompound-Related Targets

**Figure 8:**
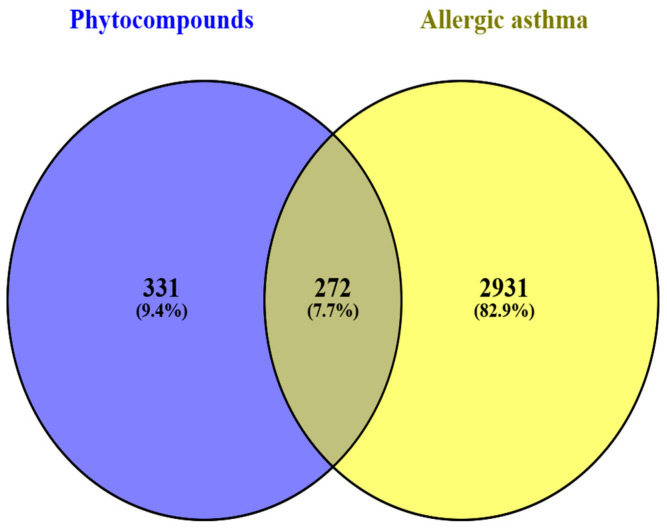
Overlapping targets of phytocompounds (603 targets) and allergic-asthma-associated targets (3200 targets).

A total of 3146 targets were identified for allergic asthma after removing duplication from public databases. The Venn diagram revealed 272 overlapping targets that were selected between 3146 targets associated with allergic asthma and 603 targets associated with phytocompounds (Figure 8).

### 2.9. Protein–Protein Interaction Network Construction

**Figure 9:**
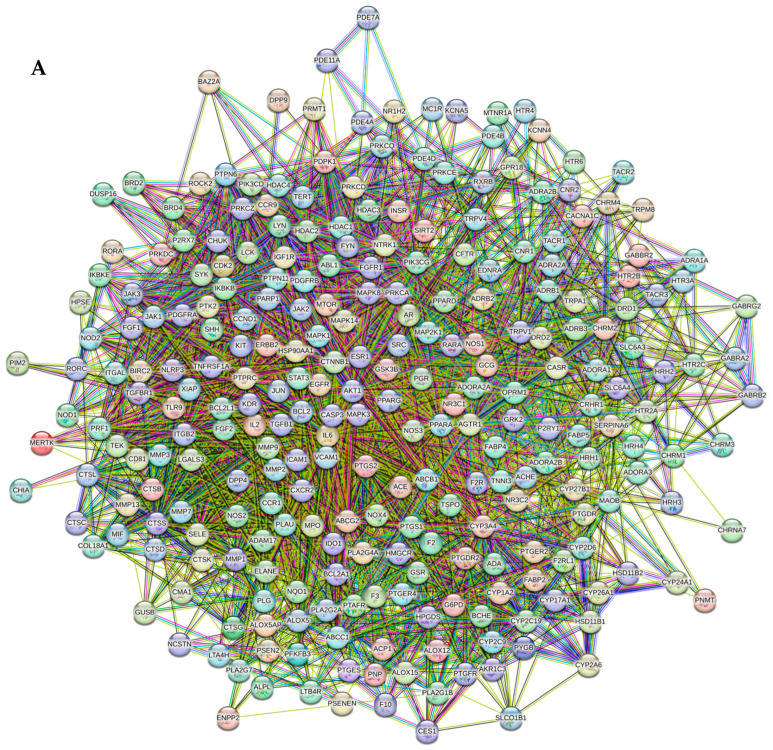

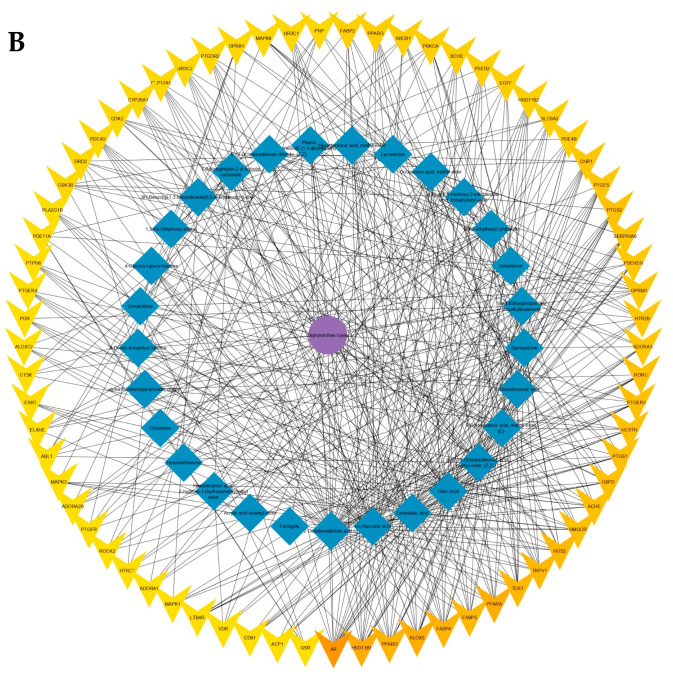
Network-pharmacology-based analysis of multi-compound and multi-target for anti-asthmatic activity. (**A**) PPI networks (270 nodes, 4505 edges) and (**B**) network diagram of compounds and with top 100 targets. The purple ellipse indicates *Z. rosea* plant, blue-green indicates the phytocompounds, and yellow nodes indicate the pathways.

From STRING analysis, 270 unique targets out of 272 overlapping targets were directly related to allergic asthma occurrence and development, indicating 270 nodes and 4505 edges (Figure 9A). Cytoscape 3.10.2 was then used to conduct PPI network analysis on the STRING network, revealing a network of interactions. In PPI networks, the IL-6 target was the highest degree (165) and was considered a hub target (Table 5). The highest degree indicates a strong correlation between the targeted genes; hence, these genes may be key targets (Figure 9B). After comparing these findings with those supplied by enrichment analysis, three genes, particularly *IL6*, *AKT1*, and *Src*, were identified as the main anti-asthmatic targets of *Z. rosea* and were chosen for molecular docking experiments.

### 2.10. Protein–Protein Interaction Network Construction and Genome Analysis

**Figure 10:**
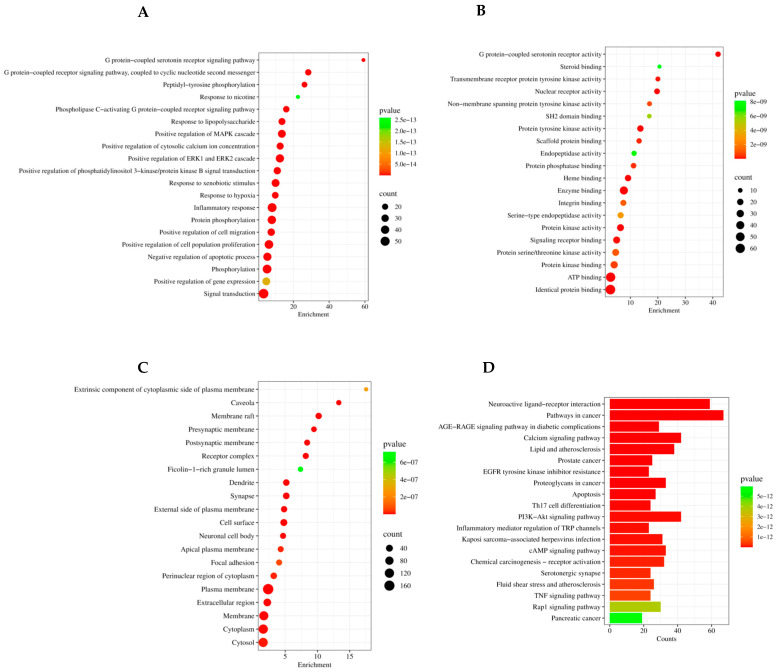
Functional annotation and potential pathways of phytocompounds for the treatment of allergic asthma in bubble graph and bar graph form, generated by SRplot. (**A**) Biological process, (**B**) molecular functions, and (**C**) cellular components. The number of genes enriched in each pathway is indicated by the size of each bubble. A larger bubble indicates a higher number of genes implicated in the pathway. The enrichment value is taken on an *x*-axis. (**D**) KEGG pathway: the top 20 items of KEGG pathway. The color of each bar represents the adjusted *p* value of each GO term. The greener the color of the term is, the larger its adjusted *p* value is in enrichment in both the bubble and bar graphs.

The PPI network connects 270 unique allergic-asthma-related targets depending on their degree and pathways. The enrichment analysis and functional annotation revealed the biological functions of *Z. rosea* targets. GO function analysis identified the biological process (BP), cellular composition (CC), molecular function (MF), and KEGG pathway entries for hub genes. KEGG pathway analysis was performed to identify the significant signaling pathways linked to the anti-asthmatic effect of *Z. rosea* (Figure 10A–D). The 20 signaling pathways from the KEGG pathway were directly connected to asthma, suggesting that these 20 signaling pathways might be the noteworthy pathways of the *Z. rosea* bulb against asthma.

### 2.11. Molecular Docking of 3 Targets

The *IL6* protein was related to two phytocompounds (9,12-octadecadienoic acid (Z,Z)-, and linoelaidic acid), the AKT1 target was related to one phytocompound (hexadecanoic acid, 2-hydroxy-1-(hydroxymethyl)ethyl ester), and the Src target was related to four phytocompounds (1-butoxypropan-2-yl isobutyl carbonate, 9,12,15-octadecatrienoic acid, methyl ester, (Z,Z,Z)-, 8H-Benzo[g]-1,3-benzodioxolo[6,5,4-de]quinolin-8-one, and daniquidone). Molecular docking was performed to verify the affinity of the target protein(s) and phytocompound(s).

### 2.12. Computational Results

**Figure 11:**
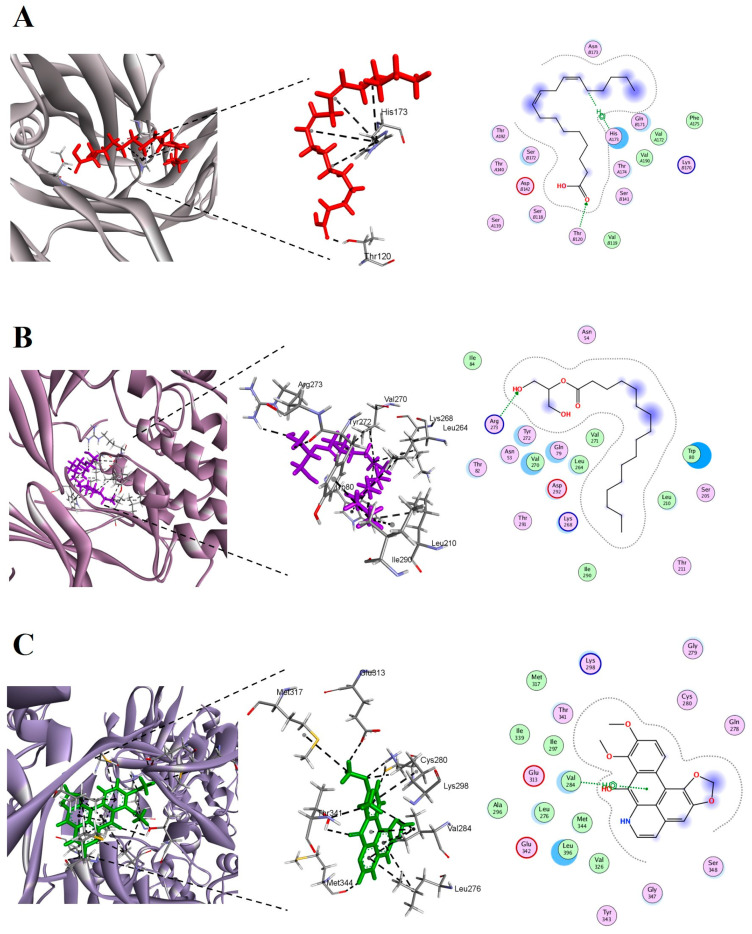
The docked complexes of three targets along with their strongest binding phytocompounds. (**A**) *IL6* docked with linoelaidic acid; (**B**) *AKT1* docked with hexadecanoic acid, 2-hydroxy-1-(hydroxymethyl)ethyl ester; and (**C**) *Src* docked with 8H-Benzo[g]-1,3-benzodioxolo[6,5,4-de]quinolin-8-one.

The docking data provide binding interactions between specific compounds, *IL6* (PDB ID: 4ZS7), *AKT1* (PDB ID: 5KCV), and *Src* (PDB ID: 6E6E), detailing binding affinity (S score) and RMSD values for each compound. 9,12-Octadecadienoic acid (Z,Z)- demonstrates a binding affinity of −6.71 kcal/mol with an RMSD of 2.74 Å, indicating a moderate binding interaction within the active site of 4ZS7. Linoelaidic acid shows a slightly stronger affinity, with an S score of −6.84 kcal/mol and an RMSD of 2.76 Å with 4ZS7, suggesting this compound may form a more stable interaction compared to 9,12-octadecadienoic acid (Table 6). The 5KCV binds stably with hexadecanoic acid, 2-hydroxy-1-(hydroxymethyl)ethyl ester. Hexadecanoic acid, 2-hydroxy-1-(hydroxymethyl) ethyl ester exhibited a strong binding affinity with an S score of −8.19 kcal/mol and an RMSD of 1.93 Å (Table 7). The 6E6E had favorable binding energy and RMSD with their compounds. The 1-butoxypropan-2-yl isobutyl carbonate displayed a docking score of −5.55 kcal/mol and an RMSD of 1.44 Å. 9,12,15-Octadecatrienoic acid, methyl ester, and (Z,Z,Z)- showed a higher affinity with an S score of −6.95 kcal/mol and an RMSD of 0.74 Å. The 8H-Benzo[g]-1,3-benzodioxolo[6,5,4-de]quinolin-8-one had the highest binding affinity with an S score of −7.18 kcal/mol and an RMSD of 1.15 Å. Daniquidone recorded an S score of −6.22 kcal/mol with an RMSD of 1.81 Å with 6E6E (Table 8). The binding score of methylprednisolone was comparable to phytocompounds with these three targets. These results exhibited that active constituents of *Z. rosea* bulbs bind stably with three target proteins and function as an anti-asthmatic agent. Molecular docking analysis demonstrated that linoelaidic acid, hexadecanoic acid (Figure 11A), 2-hydroxy-1-(hydroxymethyl)ethyl ester (Figure 11B), and 8H-Benzo[g]-1,3-benzodioxolo[6,5,4-de]quinolin-8-one (Figure 11C) showed stronger binding energies with the target protein compared to the standard drug. All the drug candidates showed hydrogen bond, Pi–pi-stacked, and van der Waals interactions with the receptor proteins.

## 3. Discussion

Asthma is a chronic respiratory condition characterized by the narrowing of the airways, leading to breathing difficulties. It affects individuals of all ages and is influenced by genetic, environmental, and immunological factors. The complex pathogenesis of asthma involves persistent inflammation and obstruction of the airways. According to the World Health Organization (WHO), approximately 235 million people worldwide were affected by asthma in 2019, with higher incidence rates observed in developed countries. However, in low- and moderate-income nations, patients often have undiagnosed and untreated asthma, highlighting the need for improved awareness and access to healthcare services [33]. It is crucial to explore novel therapeutic approaches to address the challenges posed by asthma [34].

One promising avenue of research involves the investigation of *Z. rosea* bulb extract as a potential modulator of inflammation in asthma. *Z. rosea* (family: Amaryllidaceae) is a small perennial herbaceous plant [26]. Different parts of the *Z. rosea* plant have been used in different countries for various diseases, including breast cancer, diabetes, earache, chest ailments, and viral infections [31,32]. The GC-MS analysis of the *Z. rosea* bulb methanolic extract revealed a diverse array of bioactive compounds, which may contribute to its anti-asthmatic activity.

Furthermore, treatment with *Z. rosea* extract restored hematological parameters such as the total leukocyte count (TLC), neutrophil count, and eosinophil count, as evidenced by the improvement in asthmatic scores in mice. The total leukocyte count in the blood of the treated groups was significantly lower than that in the disease group. Moreover, the hematological parameters of the experimental group treated with 600 mg/kg extract were more pronounced than those of the control group; thus, this treatment can be used as a novel drug therapy for improving the immune response in patients with asthma.

Bronchoalveolar lavage fluid (BALF) provides direct sampling of the cellular and soluble components present in the lower respiratory tract. BALF allows for the examination of various inflammatory cells, including neutrophils, lymphocytes, eosinophils, and macrophages; hence, BALF can be used to evaluate the efficacy of new drugs or treatments for lung inflammation and immune responses [35,36]. In this study, significant differences were observed among the treatment groups. Treatment with *Z. rosea* extract dose-dependently reduced TLC, neutrophil, and eosinophil counts compared to those in the diseased groups. Treatment with 600 mg/kg extract significantly affected the inflammatory parameters in the experimental group. The BALF results showed that this plant can be used to restore the immune response to allergic asthma.

Histopathological examination of lung tissues provided insight into the effects of the treatments on goblet cell morphology. Allergic asthma induction by ovalbumin led to significant alterations, including goblet cell hyperplasia and smooth muscle thickening. The standard (methylprednisolone) and *Z. rosea* 600 group mice showed significant improvement, with partial restoration of goblet cells and degenerative changes. The histopathology of the *Z. rosea* 400 group mice was markedly ameliorated, maintaining proper goblet cell arrangement and restoring smooth muscle lining. The *Z. rosea* 200 group mice exhibited pulmonary congestion and edema.

The administration of *Z. rosea* extract effectively reduced the expression of proinflammatory cytokines and the incidence of asthma compared to those in the asthmatic disease group in a dose-dependent fashion, demonstrating its potential as a therapeutic agent. Pretreatment with three different doses of *Z. rosea* extract (200, 400, or 600 mg/kg) significantly restored the levels of various cytokines, including *IL-4*, *IL-6*, *IL-10*, *IL-13*, *IL-1β*, and *TGF-β1*, in ovalbumin-induced allergic asthmatic mice compared to those in the diseased group.

Screening results for phytocompounds of *Z. rosea* bulbs by GC-MS analysis were represented, which played a decisive role in the development of allergic asthma by affecting *IL6*, *AKT1* and, *Src* genes. The associated SwissADME properties of potential compounds for different models, such as Lipinski rules, Lipinski violations, bioavailability score, and TPSA, showed positive results that strongly support the stability of phytocompounds as drug candidates.

The network pharmacology analysis related to allergic asthma outlines the process of predicting potential biological targets using public databases. These databases identified 603 potential targets for phytocompounds related to asthma, with 3146 possible targets associated with allergic asthma. A Venn diagram analysis revealed 272 matching genes, resulting in 270 unique targets relevant to both phytocompounds and allergic asthma. A protein–protein interaction (PPI) network was constructed using these targets, revealing significant hub nodes such as *IL6*, *AKT1*, *Src*, and many others. The *PI3K-Akt* pathway helps in the survival of inflammatory cells, such as eosinophils and mast cells, enhances the production of proinflammatory cytokines, regulating mucus production in airway epithelial cells, and allows for airway remodeling in allergic asthma [37]. *IL-6* is the proinflammatory cytokine that influences allergic asthma and modulates the immune response by affecting the balance between regulatory T cells and Th17 cells [38]. *Src kinases* are particularly important in the activation of various immune cells, such as mast cells and eosinophils [39]. Inhibition of these signaling pathways reduces the severity of asthma in mice.

The GO enrichment analysis of these hub nodes explored numerous biological processes (e.g., inflammatory response, phosphorylation, and signal transduction), a variety of molecular functions (e.g., nuclear receptor activity, protein tyrosine kinase activity, serine type endopeptidase activity, and protein kinase binding), and numerous cellular components (e.g., the external surface of the plasma membrane, cell surface, focal adhesion, and extracellular regions). KEGG pathways are linked to these targets, highlighting key pathways that could be therapeutically relevant in the treatment of allergic asthma (e.g., *P13K-Akt* signaling pathway, Th17 cell differentiation, *TNF* signaling pathway, and others. These findings suggested that the phytocompounds analyzed may have significant potential in influencing these pathways and could be valuable in developing treatments for allergic asthma.

Molecular docking further supported our results, indicating that there are stable binding forces between core phytocompounds and biological targets. We built a model of “herb-active compounds–targets–pathways” and found that 9,12-Octadecadienoic acid (Z,Z)-, linoelaidic acid, hexadecanoic acid, 2-hydroxy-1-(hydroxymethyl)ethyl ester, 1-butoxypropan-2-yl isobutyl carbonate, 9,12,15-octadecatrienoic acid, methyl ester, (Z,Z,Z)-, 8H-benzo[g]-1,3-benzodioxolo[6,5,4-de]quinolin-8-one, and daniquidone had a high relationship in the network, suggesting that they possess anti-asthmatic properties. Molecular docking supported our findings by verifying the interaction between phytocompounds and potential targets. Further research and clinical trials are required to examine the potential of *Z. rosea* and validate its medicinal uses, even though we have provided some interesting results.

## 4. Materials and Methods

### 4.1. Plant Material

Fresh bulbs of *Z. rosea* (1.5 kg) were bought from the local botanical market (city seeds) of Lahore, Pakistan, in August 2022. The isolate was identified and assigned a voucher number (GC. Herb.Bot.3989) by a botanist, Dr. Sohaib Muhammad, at Government College University, Lahore. The voucher specimen was deposited at the Herbarium of said institute.

### 4.2. Extraction

The bulbs were thoroughly washed with tap water to remove all the soil and dust particles and then shade-dried for 15–20 days. An electrical grinder (MHQ-86, Hangzhou, China) was used to grind the dried bulbs into a coarse powder. After that, 2.5 L of methanol was used to macerate the powder (600 g) for 7 days. The filtrate was then obtained after filtration. The procedure was repeated three times to obtain the maximum soluble material. A rotary evaporator (Lab Tech EV311 PLUS, Beijing, China) equipped with a water bath (Lab Tech EV311 PLUS, China) and recirculating chiller (Lab Tech) was used to concentrate the filtrate by evaporating the solvent at reduced pressure. The resulting semisolid extract was then dried in an oven at a temperature of 40 °C, after which it was stored in transparent glass vials. The yield of the extract was subsequently calculated [40].

### 4.3. GC-MS Analysis

The chemical composition of *Z. rosea* bulb methanolic extract was characterized by gas chromatography and mass spectrometry (GC-MS). The GC-MS analysis of *Z. rosea* bulb extract was performed on 7890A Gas Chromatograph joined to a 5975 °C mass selective detector (Agilent Technologies, Santa Clara, CA, USA), which consists of an DB-5MS capillary column (30 m long, 0.25 mm wide, and 0.25 μm film thick). Operating parameters of the mass spectrometer included relative detector gain mode, 70 eV of ionization energy, 3.3 min of filament delay time, 1666 μ/sec of scan speed, 40–550 m/z of scan range, 230 °C ion source temp, and 180 °C quadrupole temperature [41]. Helium gas (99.9%) was applied as a carrier gas, at a steady flow speed rate of 1 mL per min. The mass transfer temperature was programmed at 200 °C while injector line transfer temperature was programmed at 250 °C, with 1 μL injection volume. The oven temperature was initially programmed at 50 °C for 2 min followed by a 7 °C/min increase to 290 °C with a hold time of 37 min. A total of 1 µL of the sample was fed into the device, and its mode was split at a 5:1 ratio. Auxiliary temperature was maintained at 250 °C. Detection mode for mass spectroscopy was scanning, which ranges between 40 and 500 m/z. The mass spectra were recorded at 70 eV. The identification of the compounds was achieved by comparing obtained mass spectra of unknown peaks with those stored in the NIST (National Institute of Standards and Technology, Gaithersburg, MD, USA) [42,43].

### 4.4. Experimental Animals

Thirty-six healthy mice of either sex (25–35 g) were obtained from the animal facility of the Department of Pharmacy, University of Lahore, Lahore. These animals were divided into six equal groups, each containing six mice. The plants were kept in a controlled environment before the experiment with a 12 h light/dark cycle. The animals were provided with standard food pellets and clean water ad libitum. The mice were acclimatized to the laboratory conditions for 1 day before the experiment [44,45]. The protocol was approved by the Institutional Research Ethical Committee, the University of Lahore, Lahore, via notification IREC-2024-04.

### 4.5. Induction of Allergic Asthma

The mice were sensitized with 20 micrograms of ovalbumin (intraperitoneally) on days 0 and 14, except for the control group. After 14 days, the mice were intranasally challenged with ovalbumin for the next week (15–21 days). Phosphate-buffered saline (PBS) was given to the animals in the control group. The drug methylprednisolone was used as a standard drug [46]. The treatment protocol used is described below.

Group 1 (normal control): PBS was given to the mice in the control group.

Group 2 (disease control): The animals were fed a normal diet without any treatment.

Group 3 (positive control): The animals were treated with the standard drug methylprednisolone (15 mg intraperitoneally).

Group 4: The animals were treated with 200 mg/kg *Z. rosea* extract.

Group 5: The animals were treated with 400 mg/kg *Z. rosea* extract.

Group 6: The animals were treated with 600 mg/kg *Z. rosea* extract.

### 4.6. Blood and BALF Inflammatory Cell Count

On day 22, the mice were dissected under ether anesthesia, and blood samples were taken in ethylenediaminetetraacetic acid (EDTA) vacutainers. BALF was obtained through the bronchial route and gradually distilled with 0.5 mL of PBS before being placed in sterile Eppendorf tubes. After cytocentrifugation on a glass slide that had been fixed with methanol and stained with Wright Giemsa to count the cells according to their unique cell arrangement, the DLC in the BALF was quantified. Hematological parameters such as hemoglobin (Hb), red blood cell (RBC), platelet (PLT), and white blood cell (WBC) counts were analyzed with the help of a Sysmex XT-1800i automated hematology analyzer (Sysmex Corporation, Kobe, Japan) [47].

### 4.7. Investigation of Histopathology Slides

Formalin (Dynea, Karachi, Pakistan) (10%) was used to fix the isolated lung tissues. Sections measuring 6 µm in thickness were prepared using a microtome after the tissues were fixed in paraffin wax. Hematoxylin and eosin (H&E) (Burlington, MA, USA) were used to stain the tissues. Periodic acid–Schiff (PAS) staining (Burlington, MA, USA) was used to identify goblet cells. Inflammatory cell infiltration and goblet cell dysplasia severity were assessed by a blinded, independent histopathological examination [48].

### 4.8. Real-Time Polymerase Chain Reaction (RT–PCR)

#### 4.8.1. The mRNA Expression Levels of Cytokines

*Interleukin 4* (*IL-4*), *interleukin 6* (*IL-6*), *interleukin 1β* (*IL-1β*), *interleukin 10* (*IL-10*), *interleukin 13* (*IL-13*), and transforming growth factor beta1 (*TGF-β1*) were detected in lung tissue using RT–PCR. Total RNA was extracted from the lung tissue using the TRIzol technique, and cDNA was synthesized using a commercial kit protocol. Then, real-time PCR was performed to determine the expression levels.

#### 4.8.2. Total RNA Extraction

RNA extraction was performed from blood/tissue samples with the HiPure Total RNA Kit by Magen Biotechnology (catalog no. IVD4121, Guangdong, China). Twenty milligrams of tissue sample were immediately submerged in 4 mL of TRIzol (Invitrogen, Waltham, MA, USA), homogenized for 1 min with a tissue homogenizer, and stored at −20 °C until further processing [49].

#### 4.8.3. cDNA Synthesis and RT–PCR Amplification

For cDNA synthesis, a cDNA synthesis kit (Thermo Scientific, Waltham, MA, USA) was used. After synthesis, the cDNA was stored at −20 °C. RT–PCR was performed in a total volume of 15 µL, which included 300 ng of cDNA, primers (10 µM each), and 7.5 µL of SYBR Select Master Mix (CatNo. 4472903, Thermo Fisher Scientific—US). Reactions were run in duplicate on a SLAN-96P Real-Time PCR System (Sansure Biotech, Inc., Changsha, China) using universal thermal cycling parameters (2 min at 60 °C, 95 °C for 10 min, 40 cycles of 15 sec at 95 °C and 60 sec at 60 °C). The list of primers used is provided in Table 9.

The results were obtained using the SLAN-96P Multitasking Software Interface and analyzed using Microsoft Excel (version 2021). For gene expression quantification, the comparative C_t_ method was used. First, the gene expression levels for each sample were normalized to the expression level of the housekeeping gene, encoding glyceraldehyde 3-phosphate dehydrogenase (GAPDH) within a given sample (Δ -C_t_); the difference between the diseased samples and the treated samples was used to determine the Δ -ΔC_t_ value. The log_2_ Δ -Δ-C_t_) gives the relative fold increase in gene expression of the test versus the control condition.

### 4.9. Network Pharmacology Analysis

#### 4.9.1. Phytochemicals Database Construction and Drug Likeness Property

The phytochemicals found in *Z. rosea* bulbs were identified using GC-MS analysis. The phytochemicals identified by GC-MS were then filtered through SwissADME by Lipinski’s principles to establish their “drug-likeness” physicochemical qualities [50]. The SMILES (Simplified Molecular Input Line Entry System) phytochemicals were selected using PubChem [51].

#### 4.9.2. Compounds–Associated Target Prediction

Putative targets of GC-MS phytocompounds of *Z. rosea* were predicted using Swiss Target Prediction (STP) with the “Homo Sapiens” setting, which is based on SMILES. Swiss Target Prediction (STP) utilizes a large library of 370,000 active chemicals to suggest potential targets for over 3000 proteins from three species [50].

#### 4.9.3. Allergic Asthma—Associated Target Prediction

The allergic asthma-associated targets were retrieved from GeneCards and Omim database. The disease targets form GeneCards were selected based on scoring criteria (>0.9) and only *Homo sapiens* proteins were selected. GeneCards integrates information from various sources, including genomic, proteomic, transcriptomic, genetic, clinical, and functional data [52]. Online Mendelian Inheritance in Man (OMIM) is a comprehensive, authoritative compendium of human genes and genetic phenotypes [53].

#### 4.9.4. Venn Diagram Construction

We utilized VENNY 2.1 to identify overlapping targets between compounds of the *Z. rosea* bulb and allergic asthma compounds. This overlapping helps in identifying shared targets that may play a crucial role in the disease mechanism or therapeutic effect of the compound [54].

#### 4.9.5. Construction of Protein–Protein Interaction (PPI) Network

To create and analyze the networks for screened molecular targets, Cytoscape 3.10.2 was used. This open software platform allows for network design, visualization, analysis, the identification of target proteins and their relationships with compounds, and viewing the pathways and diseases involved [55]. We used the String database to predict protein–protein interaction (PPIs) for hub targets in *H. sapiens* [56]. The targets were reintroduced in Cytoscape to visualize the PPIs network. The most linked hubs or nodes in the network were identified and ranked on the basis of highest degree of value from PPI for hub target.

#### 4.9.6. Gene Ontology and Functional Enrichment Analysis

Gene ontology (GO) enrichment analysis was performed using the DAVID Database, which includes biological processes (BPs), cellular components (CCs), and molecular functions (MFs), and Kyoto Encyclopedia of Genes and Genomes (KEGG) pathways [57,58]. We used the SRplot platform to visualize our findings in bubble charts and bar chart format, demonstrating its usefulness as an online data analysis tool [59,60].

### 4.10. Molecular Docking

Molecular docking and scoring calculations were performed on phytocompounds identified from GC-MS analysis for their potential biological targets from *Z. rosea* bulb extract and the standard drug methylprednisolone by Molecular Operating Environment (MOE version 2019.0102) using the 3D crystal structures of the human *interleukin 6* (PDB ID: 4ZS7) at a resolution of 2.93 Å, the human RAC-alpha serine/threonine protein kinase AKT1 (PDB ID: 5KCV) at a resolution of 2.70 Å, and the human proto-oncogene tyrosine protein kinase Sr (PDB ID: 6E6E) at a resolution of 2.15 Å [61,62,63]. The structure of the protein was handled by the MOE Protonate-3D module to add missing parameters such as protons, charge atoms, and types through the AMBER99 force field. The compound structures were drawn with an MOE builder. Then, the AM1-BBC charges were calculated for each ligand, and their structures were minimized through the MMFF-94x force field until an RMS gradient of 0.1 kcal/mol/Å was achieved. The protocol was applied for the docking of six isolated compounds with 100 conformations of each compound through MOE’s default docking algorithm, i.e., the Triangle Matcher and London dG scoring functions. After docking, the best-docked conformation was selected by conformational sampling.

### 4.11. Statistical Analysis

All the results are expressed as the means of three animals ± S.D. GraphPad Prism (version 9.5.1) was used to perform the statistical analysis of the data, and *p ≤* 0.05 was considered to indicate statistical significance. One-way analysis of variance (ANOVA), followed by Dunnett’s post hoc multiple comparisons test, was applied.

## 5. Conclusions

Based on these results, it can be concluded that *Z. rosea* bulb methanolic extract contains various major classes of phytochemicals identified by GC-MS analysis, which may play a role in the treatment of respiratory disorders. In addition, the findings of this study outlined the potential therapeutic effect of *Z. rosea* bulb extract in a mouse model of ovalbumin-induced asthma. *Z. rosea* extract reportedly moderates hematological and histological parameters. These promising findings suggest that *Z. rosea* phytochemical constituents, mainly anti-asthmatic drugs, are promising candidates for further analysis of dual pneumopathological medication. The in silico and network pharmacology results suggested that the ameliorative effect of *Z. rosea* bulb may involve the *IL6*, *P13K-Akt* pathway and *Src* pathways.

## Figures and Tables

**Table 1:** The phytocompounds identified in the methanol bulb extract of *Z. rosea* identified by GC-MS analysis.

No.	Compounds	RT (mins)	Area	Area (%)	Chemical Class
1	1-Butoxypropan-2-yl isobutyl carbonate	3.393	6053538	0.72	Carbonate ester
2	1,2,4-Cyclopentanetrione, 3-methyl-	3.718	17545163	2.10	Polyketone
3	Acrylic acid isoamyl ester	4.729	9183173	1.10	Fatty acid
4	5-Hydroxymethylfurfural	6.576	8142970	0.97	Phenolic compounds
5	2-Furylglycolic acid	8.771	45694341	5.47	Phenolic compounds
6	1-tert-Butoxypropan-2-yl 3-methylbutanoate	9.690	50895896	6.09	Fatty acid ester
7	1-Isopropoxy-2,2,3-trimethyl aziridine	9.802, 9.827	58936863, 15624290	7.05, 1.87	Alkaloid
8	1,2-Epithio-3-hexanol	9.843	11749572	1.41	Alcohol
9	2-Ethyl-oxetane	9.871, 9.946	21729648, 35449679	2.60, 4.24	Terpene
10	Diallyl glycol	9.930	49736001	5.95	Phenolic compounds
11	alpha-D-Galactopyranoside, methyl	11.372	10043589	1.20	Glycoside
12	3-Deoxy-d-mannoic lactone	12.065	22446954	2.69	Lactone
13	d-Glycero-l-gluco-heptose	12.372	10916512	1.31	Carbohydrate
14	Hexadecanoic acid, methyl ester	12.727	21531557	2.58	Fatty acid
15	*n*-Hexadecanoic acid	13.309	51201496	6.12	Fatty acid
16	9-Octadecenoic acid, methyl ester, (E)-	14.570	16087543	1.92	Fatty acid
17	Haemanthamine	14.656	65354444	7.82	Alkaloid
18	9,12,15-Octadecatrienoic acid, methyl ester, (Z,Z,Z)-	14.849	5136398	0.61	Fatty acid
19	Oleic Acid	15.146	18413061	2.20	Fatty acid
20	9,12-Octadecadienoic acid (Z,Z)-	15.248	93534451	11.19	Fatty acid
21	Linoelaidic acid	15.431	6106553	0.73	Fatty acid
22	Docosanoic acid, methyl ester	17.769	1575866	0.19	Fatty acid
23	Phenol, 2,2′-methylenebis [6-(1,1-dimethylethyl)-4-methyl-	18.046	4933954	0.59	Phenol
24	Hexadecanoic acid, 2-hydroxy-1-(hydroxymethyl)ethyl ester	18.167	20460597	2.45	Fatty acid
25	Bis(2-ethylhexyl) phthalate	18.233	4950238	0.59	Alkyl esters
26	Galantamin	18.477	13325481	1.59	Alkaloid
27	Lycoramine	18.563	4486526	0.54	Alkaloid
28	4-Fluoro-5-methoxy-2-nitrobenzoic acid, trimethylsilyl ester	18.611	21714000	2.60	Phenol
29	Crinamine	18.713	13994306	1.67	Alkaloid
30	Tricyclo [6.6.0.0(3,6)]tetradeca-1(8),4,11-triene	18.775	2617101	0.31	Diterpene
31	Squalene	18.806	6705499	0.80	Triterpene
32	cis-Vaccenic acid	18.859	8191844	0.98	Fatty acid
33	9,12-Octadecadienoyl chloride, (Z,Z)-	18.900	8343481	1.00	Fatty acid
34	Estragole	18.961	4929956	0.59	Pheno
35	Crinamidine	18.988	6180864	0.74	Alkaloid
36	1,3-cis-Dihydroxycrinane	19.122	26832642	3.21	Triterpenoid
37	8H-Benzo[g]-1,3-benzodioxolo[6,5,4-de]quinolin-8-one	19.362	30788521	3.68	Phenol
38	Daniquidone	19.709	4456513	0.53	Phenol

**Table 2:** Physicochemical properties of phytocompounds for oral bioavailability and cell membrane permeability.

	Compounds	Lipinski Rules				Lipinski’s Violations	Bioavailability Score	TPSA (A^2^)
		MW	HBA	HBD	MLogP			
No.		<500	<10	≤5	≤4.15	≤1	>0.1	<140
1	1-Butoxypropan-2-yl isobutyl carbonate	232.32	4	0	1.83	0	0.55	44.76
2	1,2,4-Cyclopentanetrione, 3-methyl-	126.11	3	0	−1.02	0	0.55	51.21
3	Acrylic acid isoamyl ester	142.2	2	0	1.85	0	0.55	26.3
4	5-Hydroxymethylfurfural	126.11	3	1	−1.06	0	0.55	50.44
5	2-Furylglycolic acid	142.11	4	2	−1.1	0	0.85	70.67
6	1-tert-Butoxypropan-2-yl 3-methylbutanoate	216.32	3	0	2.27	0	0.55	35.53
7	1-Isopropoxy-2,2,3-trimethyl aziridine	143.23	2	0	1.71	0	0.55	12.24
8	1,2-Epithio-3-hexanol	132.22	1	1	1.14	0	0.55	45.53
9	2-Ethyl-oxetane	86.13	1	0	0.76	0	0.55	9.23
10	Diallyl glycol	142.2	2	0	1.04	0	0.55	18.46
11	alpha-D-Galactopyranoside, methyl	194.18	6	4	−2.4	0	0.55	99.38
12	3-Deoxy-d-mannoic lactone	162.14	5	3	−1.68	0	0.55	86.99
13	d-Glycero-l-gluco-heptose	210.18	7	6	−3.36	1	0.55	138.45
14	Hexadecanoic acid, methyl ester	270.45	2	0	4.44	1	0.55	26.3
15	*n*-Hexadecanoic acid	256.42	2	1	4.19	1	0.85	37.3
16	9-Octadecenoic acid, methyl ester, (E)-	296.49	2	0	4.8	1	0.55	26.3
17	Haemanthamine	301.34	5	1	1.32	0	0.55	51.16
18	9,12,15-Octadecatrienoic acid, methyl ester, (Z,Z,Z)-	292.46	2	0	1.32	0	0.55	26.3
19	Oleic Acid	282.46	2	1	4.57	1	0.85	37.3
20	9,12-Octadecadienoic acid (Z,Z)-	280.45	2	1	4.57	1	0.85	37.3
21	Linoelaidic acid	280.45	2	1	4.47	1	0.85	37.3
22	Docosanoic acid, methyl ester	354.61	2	0	5.79	1	0.55	26.3
23	Phenol, 2,2′-methylenebis [6-(1,1-dimethylethyl)-4-methyl-	340.5	2	2	5.05	1	0.55	40.46
24	Hexadecanoic acid, 2-hydroxy-1-(hydroxymethyl)ethyl ester	330.5	4	2	3.18	0	0.55	66.76
25	Bis(2-ethylhexyl) phthalate	390.56	4	0	5.24	1	0.55	52.6
26	Galantamin	287.35	4	1	1.74	0	0.55	41.93
27	Lycoramine	289.37	4	1	1.83	0	0.55	41.93
28	4-Fluoro-5-methoxy-2-nitrobenzoic acid, trimethylsilyl ester	287.32	6	0	1.62	0	0.55	81.35
29	Crinamine	301.34	5	1	1.32	0	0.55	51.16
30	Tricyclo [6.6.0.0(3,6)]tetradeca-1(8),4,11-triene	186.29	0	0	4.28	1	0.55	0
31	Squalene	410.72	0	0	4.28	1	0.55	0
32	cis-Vaccenic acid	282.46	2	1	4.57	1	0.85	37.3
33	9,12-Octadecadienoyl chloride, (Z,Z)-	298.89	1	0	4.57	1	0.85	17.07
34	Estragole	148.2	1	0	2.67	0	0.55	9.23
35	Crinamidine	317.34	6	1	0.87	0	0.55	63.69
36	1,3-cis-Dihydroxycrinane	289.33	5	2	1.17	0	0.55	62.16
37	8H-Benzo[g]-1,3-benzodioxolo[6,5,4-de]quinolin-8-one	335.31	6	0	1.07	0	0.55	66.88
38	Daniquidone	249.27	2	1	2.68	0	0.55	58.69

MW: Molecular Weight (g/mol); HBA: Hydrogen Bond Acceptor; HBD: Hydrogen Bond Donor; MLogP: Lipophilicity; Bioavailability Score: the ability of a drug or other substance to be absorbed and used by the body.

**Table 3:** Effect of different doses of *Z. rosea* on WBCs in BALF (mean ± S.D.).

Parameter	Unit	Normal Control	Diseased Control	Positive Control	*Z. rosea* 200	*Z. rosea* 400	*Z. rosea* 600
Lymphocytes	%	32.67 ± 0.88	72.67 ± 1.2	34.0 ± 0.58	69.32 ± 0.88	59.31 ± 0.88	57.62 ± 1.2
Monocytes	%	1.71 ± 0.33	4.72 ± 0.33	2.42 ± 0.33	3.05 ± 0.33	2.92 ± 0.58	2.79 ± 0.33

**Table 4:** The expression levels of *IL-4*, *IL-6*, *IL-1β*, *IL-10*, *IL-13*, and *TGF-β1* in normal control, disease control, positive control, and *Z. rosea* extract-treated animals.

Sample	mRNA Expression Levels of Cytokines (Mean ± S.D)					
	*IL-4*	*IL-6*	*IL-1β*	*IL-10*	*IL-13*	*TGF-β1*
Normal Control	1.153 ± 0.005	1.134 ± 0.014	0.222 ± 0.005	0.536 ± 0.006	1.770 ± 0.065	0.140 ± 0.008
Disease Control	2.917 ± 0.006	1.784 ± 0.098	5.590 ± 0.010	2.447 ± 0.061	3.489 ± 0.002	5.237 ± 0.064
Positive Control	0.577 ± 0.004	0.573 ± 0.014	0.444 ± 0.007	0.577 ± 0.019	0.155 ± 0.026	0.309 ± 0.014
*Z. rosea* 200	0.976 ± 0.007	1.390 ± 0.019	0.406 ± 0.004	0.470 ± 0.035	1.562 ± 0.029	1.308 ± 0.058
*Z. rosea* 400	0.617 ± 0.007	0.930 ± 0.051	0.105 ± 0.004	0.482 ± 0.013	0.247 ± 0.026	0.080 ± 0.017
*Z. rosea* 600	0.595 ± 0.005	0.129 ± 0.011	0.105 ± 0.006	0.535 ± 0.052	0.206 ± 0.025	0.069 ± 0.002

Normal Control: Phosphate buffer saline only. Disease Control: Normal diet without treatment. Positive Control: Methyprednicolone (15 mg/kg) intraperitoneal. *Z. rosea* 200: 200 mg/kg dose of extract was administered to mouse group. *Z. rosea* 400: 400 mg/kg dose of extract was administered to mouse group. *Z. rosea* 600: 600 mg/kg dose of extract was administered to mouse group.

**Table 5:** The degree value of top 50 PPI networks.

No	Target	Degree	No	Target	Degree
1	*IL6*	165	26	*MAPK1*	72
2	*AKT1*	150	27	*MMP2*	72
3	*SRC*	138	28	*FGF2*	70
4	*STAT3*	127	29	*KDR*	69
5	*EGFR*	119	30	*JAK2*	68
6	*CASP3*	118	31	*ACE*	67
7	*BCL2*	118	32	*MAPK14*	64
8	*PTGS2*	113	33	*KIT*	62
9	*PPARG*	106	34	*PARP1*	62
10	*TGFB1*	106	35	*NR3C1*	61
11	*MMP9*	106	36	*PRKCA*	61
12	*JUN*	105	37	*AGTR1*	59
13	*CTNNB1*	105	38	*TNFRSF1A*	59
14	*MAPK3*	104	39	*MPO*	58
15	*ESR1*	100	40	*MAPK8*	57
16	*HSP90AA1*	97	41	*PTPN11*	57
17	*ICAM1*	91	42	*IGF1R*	57
18	*IL2*	86	43	*PPARA*	56
19	*ERBB2*	85	44	*PDGFRB*	53
20	*MTOR*	82	45	*ABL1*	53
21	*PTPRC*	80	46	*FYN*	52
22	*VCAM1*	79	47	*NOS3*	51
23	*CCND1*	78	48	*PTK2*	50
24	*GSK3B*	78	49	*IKBKB*	50
25	*BCL2L1*	73	50	*HDAC1*	50

**Table 6:** Docking results of potential phytocompounds in the binding sites of *IL6*.

Compound Name	S Score (kcal/mol)	RMSD (Å)	Atom of Compounds	Atom of Receptors	Residue of Receptor	Type of Interaction Bond	Distance (Å)
*Interleukin 6* (PDB ID: 4ZS7)							
9,12-Octadecadienoic acid (Z,Z)-	−6.71	2.74	O-52 C-14	OG1 5-ring	THR 120 HIS 173	H-acceptor H-pi	2.82 4.25
Linoelaidic acid	−6.84	2.76	O-3	O	THR 174	H-donor	2.86
Methylprednisolone (Standard)	−6.29	1.69	O-1	OG1	THR 120	H-acceptor	2.83

**Table 7:** Docking results of potential phytocompounds in the binding sites of *AKT1*.

Compound Name	S Score (kcal/mol)	RMSD (Å)	Atom of Compounds	Atom of Receptors	Residue of Receptor	Type of Interaction Bond	Distance (Å)
*AKT1* (PDB ID: 5KCV)							
Hexadecanoic acid, 2-hydroxy-1-(hydroxymethyl)ethyl ester	−8.19	1.93	O-59 O-59	NE NH2	ARG 273 ARG 273	H-acceptor H-acceptor	3.09
Methylprednisolone (Standard)	−7.62	1.98	O-40 O-36 O-40	OD2 NE NH2	ASP 274 ARG 273 ARG 273	H-donor H-acceptor H-acceptor	3.23 2.98 3.14

**Table 8:** Docking results of potential phytocompounds in the binding sites of *Src*.

Compound Name	S Score (kcal/mol)	RMSD (Å)	Atom of Compounds	Atom of Receptors	Residue of Receptor	Type of Interaction Bond	Distance (Å)
*Src* (PDB ID: 6E6E)							
1-Butoxypropan-2-yl isobutyl carbonate	−5.55	1.44	O-1 C-37	NH2 6-ring	ARG 391 PHE 281	H-acceptor H-pi	2.95 4.18
9,12,15-Octadecatrienoic acid, methyl ester, (Z,Z,Z)-	−6.95	0.74	O-53	OG	SER 348	H-acceptor	3.06
8H-Benzo[g]-1,3-benzodioxolo[6,5,4-de]quinolin-8-one	−7.18	1.15	6-ring	CG1	VAL 284	pi-H	3.67
Daniquidone	−6.22	1.81	N-22 O-1	OE2 N	GLU 313 MET 344	H-donor H-acceptor	3.16 3.06
Methylprednisolone (Standard)	−6.36	1.62	O-40 O-26 O-26	OD2 NH1 NH2	ASP 407 ARG 391 ARG 391	H-donor H-acceptor H-acceptor	2.95 3.21 3.22

**Table 9:** List of primers used in reverse transcriptase polymerase chain reaction (RT–PCR).

Gene	Primer	Sequence
*IL-4*	GTACCGGGAACGGTATCCAC	Forward
	TGGTGTTCCTTGTTGCCGTA	Reverse
*IL-6*	GATGAGGCTTCCTGTCCCTACT	Forward
	TGACAGGTTTTGGAATAGCATTTCC	Reverse
*IL-10*	GGAGTCCCCATCCCAACTCA	Forward
	GCCCATAACCCCCACAACAC	Reverse
*TGF-β1*	TGATACGCCTGAGTGGCTGTCT	Forward
	TGATACGCCTGAGTGGCTGTCT	Reverse
*IL-13*	AACGGCAGCATGGTATGGAGTG	Forward
	TGGGTCCTGTAGATGGCATTGC	Reverse
*IL-1β*	GATGAGGCTTCGTGTCCCTACT	Forward
	GATGAGGCTTCGTGTCCCTACT	Reverse

## Data Availability

Data are contained within the article and Supplementary Materials.

## Supplementary Materials

The following supporting information can be downloaded at: https://www.mdpi.com/article/10.3390/ph17111558/s1, Table S1. Effect of different doses of *Z. rosea* on hematological parameters in blood (mean ± S.D.).
