# Supplementary material for: Network Pharmacology and Molecular Docking Reveal Anti-Asthmatic Potential of Zephyranthes rosea Lindl. in an Ovalbumin-Induced Asthma Model
Source: Pharmaceuticals (Basel). 2024 Nov 20;17(11):1558. doi: 10.3390/ph17111558 (PMC11597469; doi:10.3390/ph17111558)
Supplement: Supplementary file 1 [file pharmaceuticals-17-01558-s001.zip › pharmaceuticals-3222634-supplementary.pdf]

Supplementary File

Network pharmacology and molecular docking reveal anti-asthmatic potential of *Zephyranthes rosea* in an ovalbumin-induced asthma model

Amir Ali [1], Hafiz Majid Rasheed [1]*, Siddique Akber Ansari [2], Shoeb Anwar Ansari [3] and Hamad M Alkahtani [2]

[1]Faculty of Pharmacy, The University of Lahore, Lahore-54590, Pakistan
[2]Department of Pharmaceutical Chemistry, College of Pharmacy, King Saud University, P.O. Box 2457, Riyadh 11451, Saudi Arabia
[3]Department of Drug Science and Technology University of Turin.10124, Turin Italy
* Correspondence: **Author:** majid.rasheed@pharm.uol.edu.pk

Abstract: The present study aimed to evaluate the anti-inflammatory effects of *Zephyranthes rosea* in an ovalbumin-induced asthma model. Allergic asthma was induced in mice via intraperitoneal injection followed by intranasal ovalbumin challenge. The methanolic extract of *Z. rosea* (200-600 mg/kg) was orally administered to asthmatic mice for 14 days. Hematological parameters for bronchoalveolar lavage fluid (BALF) and blood were analyzed. The mRNA expression levels of interleukins and transforming growth factor-beta (TGF-β1) in lung tissues were determined using reverse transcriptase-polymerase chain reaction (RT–PCR). Analysis of network pharmacology was used to find possible *Z. rosea* targets. After building a protein-protein interaction network to find hub genes, GO and KEGG enrichment analyses were carried out to determine the potential mechanism. *In silico* analysis was performed by Molecular Operating Environment. The phytochemical and GCMS analysis of *Z. rosea* extract indicated major classes of phytochemicals. Hematological parameters in blood, and BALF from *Z. rosea* extract-treated animals were significantly reduced in a dose-dependent fashion. Histopathology revealed that *Z. rosea* had an ameliorative effect on lung tissues. Moreover, treatment with *Z. rosea* extract significantly restored the normal levels of IL-4, IL-6, IL-1β, IL-10, IL-13, and TGF-β1 in allergic asthmatic mice compared to diseased group. *In-silico* analysis, particularly of the binding affinities of *Z. rosea* phytoconstituents for IL6, AKT1, and Src, supported *in-vivo* results. These findings indicated that *Z. rosea* extract significantly ameliorates cellular and molecular biomarkers of bronchial inflammation and could be a potential candidate for treating allergic asthma.

Keywords: Asthma; Ovalbumin; Bronchoalveolar lavage fluid; Interleukin 6; Akt Serine/Threonine Kinase 1; Proto-oncogene tyrosine-protein kinase Src; Network pharmacology; Tumor necrosis factor-alpha; *Zephyranthes rosea*

Table S1. Effect of different doses of *Z. rosea* on hematological parameters in blood (mean ± S.D.).

Parameter	Unit	Normal Control	Diseased Control	Positive Control	*Z. rosea* 200	*Z. rosea* 400	*Z. rosea* 600
**Hb**	g/dL	14.9 ± 0.058	13.21 ± 0.152	13.95 ± 0.08	12.85 ± 0.088	14.21 ± 0.12	14.97 ± 0.088
**RBCs**	$\times 10^6/\mu L$	8.95 ± 0.117	7.21 ± 0.09	7.91 ± 0.216	7.29 ± 0.35	8.26 ± 0.05	8.81 ± 0.03
**Platelets**	$\times 10^3/\mu L$	571.0 ± 18.77	1071 ± 26.72	821.31 ± 17.05	989.12 ± 12.71	923.31 ± 4.98	891.62 ± 12.72
**Lymphocytes**	%	42.24 ± 1.45	79.12 ± 4.16	51.0 ± 0.578	62.32 ± 1.76	61.21 ± 1.2	58.51 ± 1.45
**Monocytes**	%	3.27 ± 0.88	6.0 ± 0.58	3.91 ± 0.167	4.12 ± 0.58	3.82 ± 0.33	3.59 ± 0.58
